# Quantitative evaluation of nonlinear methods for population structure visualization and inference

**DOI:** 10.1093/g3journal/jkac191

**Published:** 2022-07-28

**Authors:** Jordan Ubbens, Mitchell J Feldmann, Ian Stavness, Andrew G Sharpe

**Affiliations:** Global Institute for Food Security (GIFS), University of Saskatchewan, Saskatoon, SKS7N 0W9, Canada; Department of Plant Sciences, University of California, Davis, CA95616, USA; Global Institute for Food Security (GIFS), University of Saskatchewan, Saskatoon, SKS7N 0W9, Canada; Department of Computer Science, University of Saskatchewan, Saskatoon, SKS7N 0W9, Canada; Global Institute for Food Security (GIFS), University of Saskatchewan, Saskatoon, SKS7N 0W9, Canada

**Keywords:** population structure, visualization, machine learning

## Abstract

Population structure (also called genetic structure and population stratification) is the presence of a systematic difference in allele frequencies between subpopulations in a population as a result of nonrandom mating between individuals. It can be informative of genetic ancestry, and in the context of medical genetics, it is an important confounding variable in genome-wide association studies. Recently, many nonlinear dimensionality reduction techniques have been proposed for the population structure visualization task. However, an objective comparison of these techniques has so far been missing from the literature. In this article, we discuss the previously proposed nonlinear techniques and some of their potential weaknesses. We then propose a novel quantitative evaluation methodology for comparing these nonlinear techniques, based on populations for which pedigree is known a priori either through artificial selection or simulation. Based on this evaluation metric, we find graph-based algorithms such as t-SNE and UMAP to be superior to principal component analysis, while neural network-based methods fall behind.

## Introduction

Population structure—the patterns of ancestral similarities and dissimilarities with arbitrarily defined populations—is a topic of primary concern in population, quantitative, and evolutionary genetics in humans, plants, microbes, and animals. The basic cause of population structure in sexually reproducing species is nonrandom mating between groups: if all individuals within a population mate randomly, then the allele frequencies should be similar between groups. Population structure commonly arises from physical or reproductive separation and isolation by distance, barriers, like mountains and rivers, followed by genetic drift (Holsinger and Weir 2009; Petkova *et al.* 2016). Other causes include gene flow from migrations, population bottlenecks and expansions, founder effects, evolutionary pressure, random chance, and (in humans) cultural factors. As such, it should be expected that clusters, such as families, tribes, and clades should appear naturally in the data (Holsinger and Weir 2009; Battey *et al.* 2021).

Estimates of population structure are usually derived from linear factor models such as principal component analysis (PCA), but nonlinear dimensionality reduction techniques have attracted interest in recent literature. Estimation of population structure is also critical for our ability to accurately link genetic variation to phenotypic variation, because population structure can be a major confounding factor in genome-wide association studies (GWAS) (Lander and Schork 1994; Pritchard and Donnelly 2001; Freedman *et al.* 2004; Marchini *et al.* 2004; Yu *et al.* 2006). Dimensionality reduction has been an important tool for geneticists and has been widely used both to control for the effects of population structure in GWAS (Patterson *et al.* 2006; Price *et al.* 2006; Yu *et al.* 2006) and visualization and inference of genetic variation, i.e. population structure (Steinig *et al.* 2016; Francis 2017; Marnetto and Huerta-Sánchez 2017; Battey *et al.* 2021).

One existing challenge with estimating population structure is that there is no ground truth with which to compare. Sometimes, geographic distances are used as correlates of Euclidean distance in principal component space to measure population reconstruction and representation (Battey *et al.* 2021). Given patterns of limited dispersal, on average, in many natural species, it would make sense that the genetic distance would be correlated with measures of geographic distance (Van Heerwaarden *et al.* 2011; Battey *et al.* 2021). However, for many locally restricted species and populations, e.g. breeding programs, geography is not a correct ground truth metric by which to judge tools for population structure visualization and inference.

Here, using pedigreed populations as well as simulations, we deploy a ground-truthing method for comparing and contrasting dimensionality reduction (embedding) techniques for population structure visualization and inference. We simulate populations with or without assortative mating (selection) and with or without migration (population structure) and keep track of the pedigree, subpopulation membership, and individual level genotypes over multiple generations. We track the length of the shortest path between individuals in the pedigree network as the ground truth metric for ancestral distance. Pairs of individuals across more distantly related subpopulations will have a greater number of edges between them, compared to pairs of individuals within subpopulations, because they share a more distant most recent common ancestor. We use this ground truth to compare PCA, t-SNE, UMAP, an autoencoder (AE), a variational autoencoder (VAE), contrastive embedding learning, and random projections. In these experiments, UMAP and t-SNE significantly outperform all other methods regardless of mating, selection, or structure.

### Review of existing methods

There are several nonlinear models for population structure visualization and inference, which have been demonstrated in the literature. These methods can generally be divided into 3 categories: graph-based algorithms, AEs, and VAEs. Here, we briefly discuss these algorithms and their applications.

In addition to the methods we review below, there are many application-specific algorithms, which have been proposed in the literature, including AWClust, SHIPS, NETVIEW, iNJclust, and others (Alhusain and Hafez 2018). These methods mostly involve performing PCA followed by a secondary clustering step. Although these could also be useful techniques, in the present work, we focus our analyses on the major classes of general-purpose nonlinear dimensionality reduction methods, which have been applied to population genetics in recent literature.

#### Principal component analysis

While nonlinear methods have seen increasing interest in recent years, population structure is still most often analyzed using a linear method called PCA. PCA uses eigenanalysis to produce a number of vectors, called principal components, where each component captures the axis of maximum variance in the data while being orthogonal to the previous components. Using these vectors, one can calculate a linear transformation of the data, which casts the samples into a lower-dimensional space while representing the maximum amount of linear variance. Although it is not capable of representing nonlinear relationships, PCA has the advantage of being more interpretable than nonlinear methods as the axes of the visualization can be interpreted in this way.

#### Multidimensional scaling

Multidimensional scaling (MDS) is a foundational method in distance-based dimensionality reduction which takes a direct approach to compacting high-dimensional data into a low-dimensional space. First, the pairwise distances between each pair of samples in the original space are calculated. Next, a projection to the low-dimensional space is learned by minimizing the differences between the pairwise distances in the low-dimensional space and the calculated distances in the original space. MDS has largely been superseded by other distance-based methods such as t-SNE and UMAP, which aim to represent structures of local connectedness in the data.

#### Graph-based algorithms

Two of the most popular techniques used for visualizing high-dimensional data, which have both seen application in population genetic data, are t-SNE (Platzer 2013; Li *et al.* 2017) and UMAP (Diaz-Papkovich *et al.* 2019). These methods are part of a family of techniques, which are concerned with representing the data as a graph with various edge lengths. These techniques aim to reconstruct the graph in a lower-dimensional space, while maintaining the topological structure from the original high-dimensional space. This means that samples that are neighbors in the input space will tend to form neighborhoods in the output space.

#### Autoencoders

An AE is a type of neural network that is commonly used for learning a set of features from data in an unsupervised fashion. Recently, AEs have been applied to population genetic data (López-Cortés *et al.* 2020; Ausmees and Nettelblad 2022). AEs are comprised of 2 components, an encoder neural network and a decoder neural network, which jointly reconstruct an input:

(1) $$\hat{x}=f_{\text{decoder}}\left(f_{\text{encoder}}(x)\right).$$

The encoder network *f*_encoder_ compresses the high-dimensional input **x** into a lower-dimensional space, while the decoder network *f*_decoder_ outputs an approximation $\hat{x}$ of the original input using only the low-dimensional embedding provided by the encoder. Since the embedding space acts as a bottleneck, it must efficiently represent as much of the relevant information as possible from the original signal. For this reason, AEs have often been used as a means of extracting informative and discriminative features for downstream tasks, where the embedding representation can be re-used for another purpose. Specialized AEs can also be used for tasks such as denoising input data, like images (Vincent *et al.* 2008).

#### Variational autoencoders

The VAE is a latent variable model that defines a generative model over data (Kingma and Welling 2014). VAEs have been used for population data using both the standard Gaussian prior (Battey *et al.* 2021), as well as a Gaussian mixture prior (GMM-VAE) (Meisner and Albrechtsen 2020). When learning a latent variable model such as a VAE, one would like to maximize the probability of the data under the model:

(2) $$p(x)=\int p_{\theta }(x|z)p(z)dz$$

where **z** is the latent variable and *θ* are the model parameters. In this case, performing exact inference is analytically intractable due to the integral in Equation (2). However, we are able to minimize the *evidence lower bound* (ELBO) directly, given by

(3) $$\begin{array}{c} \;\text{log}\;\left(p_{\theta }(x,z)\right)=\text{log}\;\left(\int p_{\theta }(x|z)p(z)dz\right)\\ =\text{log}\;\left(E_{q}\left[p_{\theta }(x|z)\frac{p(x)}{q(z)}q(z)\right]\right)\\ \geq E_{q}\left[\text{log}\;\left(\frac{p_{\theta }(x,z)}{q(z)}\right)\right]\\ =E_{q}\left[\text{log}\;\left(\frac{p(z)}{q(z)}\right)\right]+E_{q}\left[\text{log}\;\left(p_{\theta }(x|z)\right)\right], \end{array}$$

where *q* is the tractable prior. VAEs are trained using stochastic sampling, which we will skip here for brevity, and are described in more detail in Kingma and Welling (2014). VAEs and their many variants are useful models because the posterior density of the model approximates the tractable prior. This means that sampling new data from the complex, multi-modal data distribution is as simple as sampling from the known prior. It also means that, unlike with the AE, the density of the posterior is approximately continuous in the latent space and so it is often possible to smoothly interpolate between points in this space.

### Challenges with existing nonlinear methods

#### Nonlinear methods distort distances

AEs, VAEs, and variants use decoders, which are parameterized by neural networks. It is well-established that, when the decoder is a nonlinear function, the Euclidean distance between points in the embedding space is meaningless, as these distances are distorted by the decoder (Arvanitidis *et al.* 2018). The amount of instantaneous distortion is given by the Jacobian of the decoder evaluated with respect to **z**. This is straightforward to intuit: imagine moving through the embedding space while monitoring the output of the decoder. There are areas of the space where moving a short distance in a straight line elicits large changes in the output of decoder, and other areas where moving even a large distance does not change the output of the decoder at all. This poses a problem when using these methods for visualization and inference, as it is natural to interpret distance between points in the visualization in terms of Euclidean distance, without knowing that the data actually lie on some nonlinear submanifold embedded in the space.

While neural methods distort distances arbitrarily (Arvanitidis *et al.* 2018), it is important to note that just because a nonlinear decoder is absent does not mean that Euclidean distance is always respected. All nonlinear methods will distort the local and global structure of the data, meaning that distances between and within clusters are inconsistent and can change with different hyperparameters (Kobak and Berens 2019). In addition, some methods, such as t-SNE, prefer nonlinear (e.g. t-distributed) distance for a specific visualization purpose, for example to emphasize local differences and avoid the over-crowding of points in a local area. Although nonlinear methods attempt to preserve certain esthetic characteristics of the data, practitioners should be aware that different methods will distort distance in different ways, and this limits interpretability.

#### VAEs are sensitive to the choice of prior

The VAE is a powerful generative model. However, the inference model $q_{\theta }(z|x)$ learned by the VAE is generally meaningless with respect to the true data generating distribution. The ELBO is only a lower bound on the model evidence, and the difference between the lower bound and the true model evidence is given by

(4) $$\begin{array}{c} \;\text{log}\;\left(p(x)\right)-\text{ELBO}=D_{\text{KL}}(q(z)||p(z|x)). \end{array}$$

Therefore, the model is an unboundedly bad approximation of the true data distribution, where the quality of that approximation is given by the Kullback–Leibler divergence between *q* and the true distribution. Simply put, the samples will be approximately distributed according to whatever prior is chosen, regardless of what the actual underlying data generating distribution is. Using an implicit prior, one could even make the samples take on arbitrary distributions (Huszár 2017). For this reason, if the model will be used for making inferences about population structure, then the user must ensure that the chosen prior accurately represents the true underlying distribution, making the lower bound tight.

#### Nonlinear methods may result in spurious clustering

Population stratification naturally results in uneven distribution of alleles, but not necessarily completely discrete, compact clusters. For example, gradual migration over time may result in more of a gradient of allele frequency than in *K* completely distinct groupings. Nonlinear methods may exaggerate the presence of clusters, when the actual distribution of alleles is less discrete. This is true of methods that explicitly assign samples to clusters, such as GMM-VAE, as well as distance-based methods, such as t-SNE and UMAP, which may imply structure and continuous relationships in the data that do not exist (Kobak and Berens 2019).

## Materials and methods

### Evaluation using pedigreed populations

The objective evaluation of visualization methods is challenging. Given several options, practitioners may disagree on which visualization is subjectively the best for a given dataset. Battey *et al.* (2021) proposed to quantify the accuracy of a visualization by comparing the Euclidean distance between samples with their corresponding geographic distance. This is a good solution under the assumption that a correlation exists between geographic distance and distance in ancestry, which is generally true for any population where migration occurs. We extend this line of thinking by using pedigreed populations where ancestry can be calculated exactly, using the number of generations between ancestors and descendants. If ancestry is being represented well in the visualizations, then this distance should correlate with the Euclidean distance between the individuals in the embeddings. When the distance between ancestors and descendants is accurately modeled, the distance between, for example, siblings, is also enforced as they are anchored by their respective parents. In total, we use 4 publicly available datasets of genotyped individuals that include pedigree information.

#### Strawberry (*Fragaria* sp.)

We analyzed the global pedigree of wild and cultivated strawberry (*Fragaria* sp.) reported by Pincot *et al.* (2021). These pedigree records were assembled for 8,851 individuals, including 2,656 cultivars developed since 1775. SNP marker genotypes for 1,495 individuals were available, including 1,235 UCD and 260 USDA accessions (asexually propagated individuals) previously genotyped by Hardigan *et al.* (2018) with the iStraw35 SNP array (Bassil *et al.* 2015; Verma *et al.* 2016). This pedigree extends 23 generations for 2 ascendant-descendent pairs and has a median depth of 7 generations. The strawberry dataset included both natural accessions and Californian breeding material; the majority of samples are from the latter and the former includes diverse samples from Asia, Europe, North America, and South America. Despite this, Pincot *et al.* (2021) suggested a high level of admixture and allele sharing among the individuals in the metropolitan population.

#### Pig (*Sus scrofa*)

We analyzed a pig dataset that PIC (a Genus company) made available for comparing genomic prediction methods by Cleveland *et al.* (2012). The dataset contains 3,534 individuals with 52,842 SNP genotypes from the Illumina PorcineSNP60 chip (Ramos *et al.* 2009) and a pedigree including parents and grandparents of the genotyped animals (Cleveland *et al.* 2012). This pedigree extends 14 generations for 417 ascendant-descendant pairs and has a median depth of 7 generations. The PIC Pig population is strictly a breeding population and we expect it to be without substantial population structure or well defined subpopulations.

#### Soay sheep (*Ovis aries*)

We analyzed the wild Soay sheep population reported by Stoffel *et al.* (2021). This dataset includes genetypes from the Illumina Ovine SNP50 BeadChip containing 51,135 SNP markers for 5,952 wild Soay sheep with both genotypes and pedigree. There are 8,172 entries in the pedigree records. This pedigree extends 13 generations for 1 ascendant–descendant pair and has a median depth of 5 generations. The Soay sheep is a natural population in which ascendants and descendants are genotypes and pedigreed and we expect there to be limited population structure or well defined subpopulations.

#### Florida Scrub-Jay (*Aphelocoma coerulescens*)

We analyzed a natural Florida Scrub-Jay population from Chen *et al.* (2019). Our final pedigree consists of 6,936 individuals with truncated birth years post-1990 with SNP genotypes of 10,731 autosomal SNPs from 3,404 individuals (Chen *et al.* 2019). This pedigree extends 8 generations for 54 ascendant–descendent pairs and has a median depth of 2 generations. The Florida Scrub-Jay population is a wild population including genotypes for ascendants and descendants and we expect there to be limited population structure or well-defined subpopulations. Chen *et al.* (2019) noted that the genetic contribution of immigrants to be 75% in the final population, which suggests a high degree of admixture.

### Evaluation using simulation

**Fig. 1.**
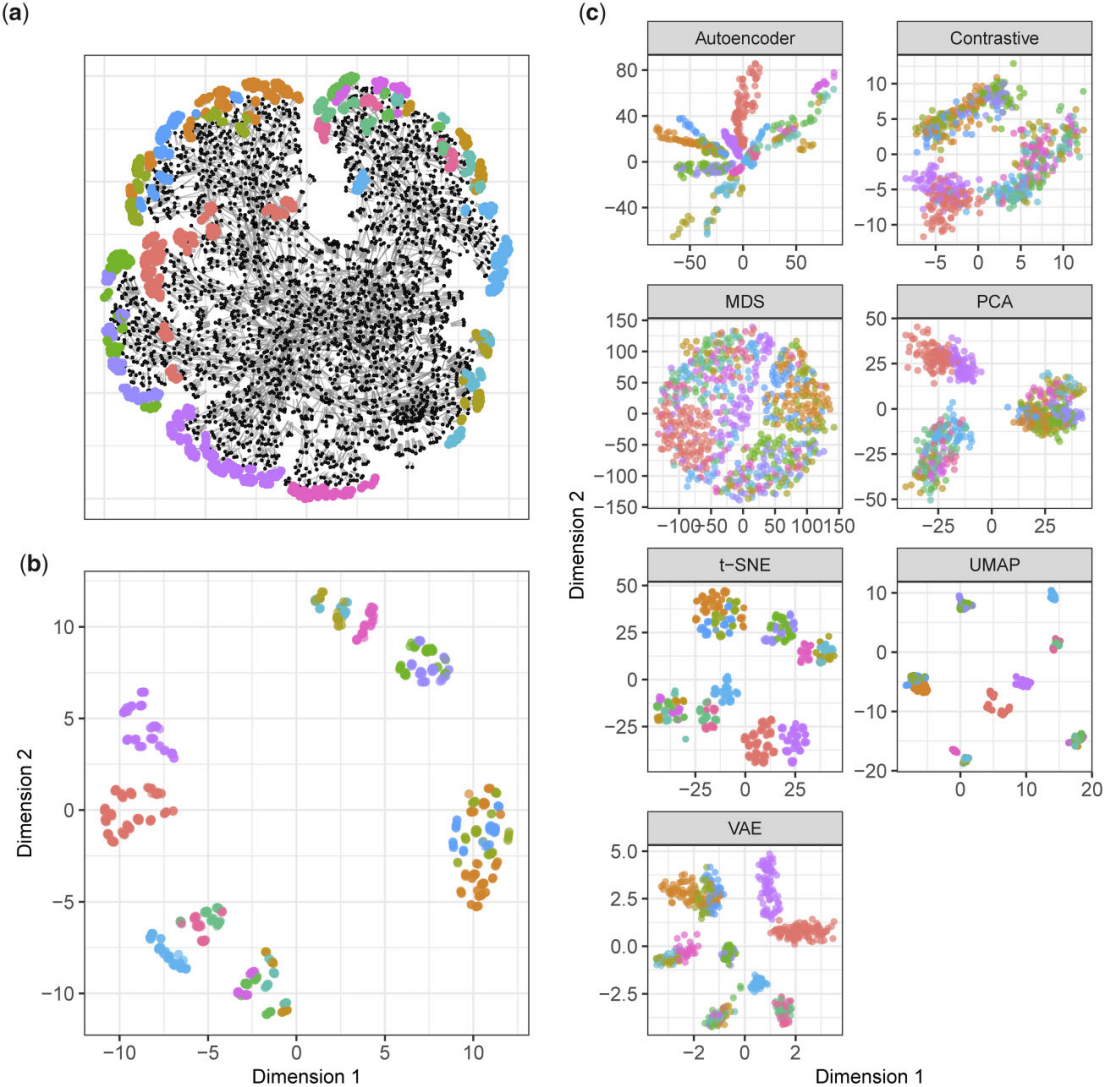
Using true genealogy (pedigree) as the truth to compare projection methods. In each plot, colors correspond to different subpopulations resulting from simulated migrations. Points are plotted in a random order. a) An example of a simulated population with migration and random mating showing ascendants (black nodes), genotyped descendants (colored nodes), and relationships (gray edges). b) The “ground-truth” pairwise distances for the individuals of the last generation in all subpopulations, as calculated from the family tree, were used as the difference matrix in MDS. This provides an example of what a visualization might look like if the distances between points represented differences in ancestry as accurately as possible. c) Candidate visualizations of the simulated population.

To provide a complete ground truth for evaluation, which includes known pairwise ancestral distances between both distantly and immediately related individuals, we use a variety of population-level simulations. These simulations are based on the SeqBreed software package (Pérez-Enciso *et al.* 2020), which was modified to begin from a hypothetical founder population with a uniformly random distribution of alleles. This ensures that all of the population structure present in the data is from the simulation. An example of one such simulated population is shown in Fig. 1.

Each simulation started from a diploid founding population of 100 individuals and proceeded for 10 generations. A constant recombination rate of 1 cM/Mbp is assumed, and no sex chromosomes were simulated. In total, 24,000 biallelic SNPs were simulated in each population and were used to genotype the terminal nodes (extant individuals).

We performed simulations with migration and without migration. For the simulations with migration, half of the current generation emigrates to found a separate subpopulation with some probability (*P = *0.3). For each of the simulations with and without migration, we performed versions with random and assortative mating.

For the simulations with random mating, every individual within the current generation pairs with a random other individual. A random number of offspring, between 1 and 4, are produced. This resulted in an average *F*_ST_ = 0.083, 0.24≤MAF≤0.5, which is concordant with low genetic differentiation between the known subpopulations, expected in a stable, random mating population. For simulations with assortative mating, we defined a hypothetical quantitative trait with 10 randomly selected QTN ($h^{2}=0.5$). For each generation, the top 50% of the individuals are selected based on their phenotype, and each pairing produces between 2 and 8 offspring. This resulted in an average *F*_ST_ = 0.155, 0≤MAF≤0.5, which is concordant with moderate genetic differentiation between the known subpopulations. The populations are twice as differentiated.

**Fig. 2.**
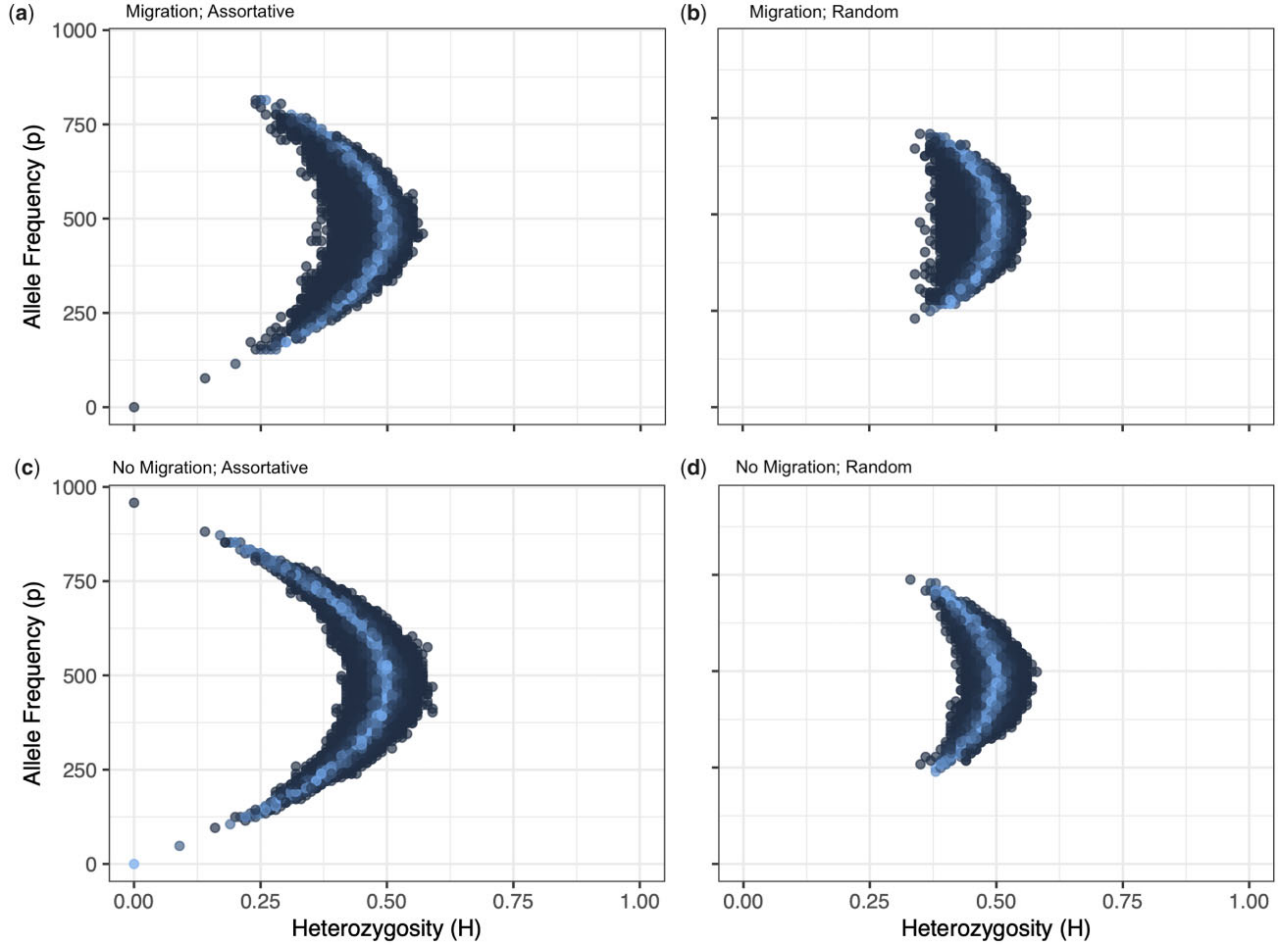
Allele frequency spectrum in a representative simulation with migration (a, b) and no migration (c, d) with assortative (a, c) and random (b, d) mating. Color represents the $\chi^{2}$ deviations from expected frequency with lighter colors representing larger *P*-values (less deviance). As expected, populations exposed to selective pressure (i.e. mating or migration) had larger deviation and more extreme allele frequencies than populations exposed to fewer selective pressures.

Simulations were run 100 times each to obtain error estimates. Population genetic parameters are estimated using *popgen()* from snpReady v0.9.6 (Granato and Fritsche-Neto 2018) in R v4.2.0 (R Core Team 2021). The allele frequency spectrum as a function of heterozysosity for each site is shown in Fig. 2 (Ferretti *et al.* 2018). Expected identity by descent (IBD) matrices were calculated from the known pedigree in each simulated population using *Amatrix()* from AGHmatrix v2.0.4 (Amadeu *et al.* 2016) in R.

### Models evaluated

For all evaluations, we compare PCA, t-SNE, UMAP, MDS, an AE, a VAE, and a learned embedding obtained via an unsupervised contrastive technique (Ye *et al.* 2019). We also use a random nonlinear projection from a randomly initialized 2-layer neural network as a comparison baseline. We use the umap-learn implementation for UMAP (McInnes *et al.* 2018) and the scikit-learn implementation for t-SNE and MDS (Pedregosa *et al.* 2011). In each case, the marker data were used as the input directly. Simulation output from SeqBreed represents alleles as 0 or 1. The biallelic sites were used directly for input without centering. In the case of the VAE, the SNP data were normalized to [0,1] due to the cross-entropy term in the loss function. When using techniques such as t-SNE and UMAP, it is common to preprocess the data using PCA and use the first *n* principal components as the input. However, we found that for the pedigree distance metric, not preprocessing the data in this way provider better performance.

Since, in practice, the ancestral relationships are unknown, it is not realistic to tune each method specifically to maximize performance on the pedigree distance task. This means that there is no principled way to select hyperparameters or architectures for each method, as in real-world use cases there will be no good criteria for which resulting visualizations are better than others. For this reason, we use the default hyperparameters for UMAP and t-SNE. For the neural network-based methods, we select hyperparameters that allow the loss to decrease to a plateau, indicating that, at the very least, the model has fit the data to some extent without catastrophic problems in training.

For the AE and contrastive methods, we use fully connected encoders and decoders with 2 hidden layers (256 and 128 units) and a learning rate of $10^{-4}$. For the AE, we used categorical cross-entropy as the reconstruction loss. We use the Adam optimizer (Kingma and Ba 2015) in all cases and train to 100 epochs because this was a single number which allowed the loss for all of the neural methods to plateau for all of the datasets.

For the VAE, we use the architecture described in Battey *et al.* (2021), with the exception of batch normalization used in the encoder and decoder. While Battey *et al.* (2021) observed worse performance with batch normalization, it improved the performance of the VAE in our tests and stabilized training, allowing the VAE to avoid posterior collapse in some cases (Bowman *et al.* 2016). We used a learning rate of $10^{-3}$ and (following Battey *et al.* 2021) binary cross-entropy for the log likelihood term.

In Battey *et al.* (2021), the authors described an issue that they refer to as overfitting. The authors proposed to remedy this problem by using early stopping based on held-out samples. We found early stopping to be less important for the VAE; however, with some datasets, the performance did show small fluctuations over the number of training epochs. We decided to not include early stopping in our experiments, as holding out data for early stopping means that it is impossible to compare the method to others as it does not take into account all of the data.

Unsupervised embedding learning is a commonly used technique for performing tasks such as information retrieval in unlabeled datasets. These techniques attempt to learn an embedding space where similar samples lie close together in the space and dissimilar samples are farther apart. For our purposes, we chose to use a contrastive embedding learning technique with instance-wise supervision (Ye *et al.* 2019). Training samples **x** are augmented to create positive samples $\hat{x}$. This is done by randomly changing homozygous sites to heterozygous with some probability (*P = *0.1). We then minimize the loss function:

(5) $$-\text{log}\;\frac{e^{\phi (x,\hat{x})}}{e^{\phi (x,\hat{x})}+e^{\phi (x,w)}},$$

where ϕ is a similarity metric, here we use the cosine similarity, and **w** is a different sample than **x**. We found that cosine similarity performs better on the pedigree distance metric than other similarity metrics, such as inverse Euclidean distance.

## Results and discussion

For the first experiments, we report Pearson’s correlation between the Euclidean distance in the visualization and the known ancestral distance (as given by the pedigree records or the simulation). We provide 2 sets of results, based on 2 potential interpretations of how distance between ancestors should be portrayed visually. The upper portion of Table 1 shows results where the simple Euclidean distance between points in the visualization is used, while the lower portion of Table 1 shows results where ${\;\text{log}\;}_{2}$ of this distance is used. The former is based on the assumption that pedigree distance should be represented linearly, independent of depth, while the latter assumes that distance between points should correlate with the proportion of ancestry represented by that ancestor. For example, if an individual is separated from their parent by a distance of 4 units, then the linear distance assumes that their grandparent should be 8 (2 × 4) units away, while the exponential distance assumes that the grandparent should be 16 (4^2^) units away. We found that, in general, the ${\;\text{log}\;}_{2}$ definition resulted in higher correlations with marker inferred distance.

**Table 1.** Pearson correlation (*r*) between the Euclidean distance, *D*, and ${\;\text{log}\;}_{2}$ of the Euclidean distance, ${\;\text{log}\;}_{2}(D)$, in the visualization and the known pedigree distance.

Distance metric	Model	Strawberry	Pig	Soay sheep	Florida Scrub-Jay	Migration		No migration	
						Random	Assortative	Random	Assortative
*D*	UMAP	0.85	0.73	**0.78**	**0.18**	**0.80** (± 0.19)^*g*^	**0.78** (± 0.17)^*e*^	**0.16** (± 0.02)^*f*^	**0.24** (± 0.03)^*f*^
	t-SNE	0.76	**0.77**	0.75	0.17	0.72 (± 0.20)^*f*^	0.74 (± 0.18)^*d*^^,^^*e*^	0.11 (± 0.01)^*d*^	0.19 (± 0.03)^*d*^
	PCA	**0.86**	0.48	0.07	0.15	0.76 (± 0.21)^*f*^^,^^*g*^	0.75 (± 0.20)^*d*^^,^^*e*^	0.11 (± 0.02)^*d*^	0.18 (± 0.04)^*d*^
	VAE	0.60	0.52	0.26	0.16	0.64 (± 0.21)^*e*^	0.68 (± 0.16)^*d*^	0.13 (± 0.02)^*e*^	0.22 (± 0.04)^*e*^
	Contrastive	0.73	0.54	0.12	0.16	0.49 (± 0.21)^*c*^	0.52 (± 0.22)^*c*^	0.05 (± 0.01)^*c*^	0.11 (± 0.03)^*c*^
	AE	0.76	0.44	0.01	0.14	0.35 (± 0.16)^*d*^	0.35 (± 0.18)^*b*^	0.03 (± 0.01)^*b*^	0.05 (± 0.03)^*b*^
	MDS	0.53	0.26	0.02	0.14	0.20 (± 0.15)^*b*^	0.46 (± 0.19)^*c*^	0.02 (± 0.00)^*a*^	0.05 (± 0.02)^*b*^
	Random	0.06	0.07	0.11	0.10	0.04 (± 0.03)^*a*^	0.07 (± 0.05)^*a*^	0.02 (± 0.01)^*a*^	0.03 (± 0.01)^*a*^
${\;\text{log}\;}_{2}(D)$	UMAP	0.71	**0.72**	**0.70**	0.29	**0.85** (± 0.16)^*f*^	**0.82** (± 0.12)^*d*^	**0.28** (± 0.02)^*g*^	**0.39** (± 0.03)^*g*^
	t-SNE	0.64	0.70	0.64	**0.31**	0.75 (± 0.19)^*e*^	0.78 (± 0.15)^*c*^^,^^*d*^	0.19 (± 0.02)^*f*^	0.30 (± 0.03)^*f*^
	PCA	**0.74**	0.57	0.14	0.16	0.74 (± 0.20)^*e*^	0.74 (± 0.19)^*c*^	0.10 (± 0.02)^*d*^	0.17 (± 0.04)^*d*^
	VAE	0.56	0.55	0.32	0.16	0.66 (± 0.19)^*d*^	0.71 (± 0.14)^*c*^	0.17 (± 0.02)^*e*^	0.28 (± 0.04)^*e*^
	Contrastive	0.57	0.56	0.12	0.17	0.50 (± 0.21)^*c*^	0.52 (± 0.21)^*b*^	0.05 (± 0.01)^*b*^	0.11 (± 0.03)^*c*^
	AE	0.50	0.33	0.21	0.15	0.52 (± 0.17)^*c*^	0.48 (± 0.14)^*b*^	0.07 (± 0.02)^*c*^	0.11 (± 0.03)^*c*^
	MDS	0.48	0.25	0.02	0.01	0.20 (± 0.14)^*b*^	0.47 (± 0.20)^*b*^	0.02 (± 0.00)^*a*^	0.05 (± 0.02)^*b*^
	Random	0.22	0.10	0.12	0.07	0.03 (± 0.02)^*a*^	0.07 (± 0.05)^*a*^	0.02 (± 0.01)^*a*^	0.03 (± 0.01)^*a*^

**Fig. 3.**
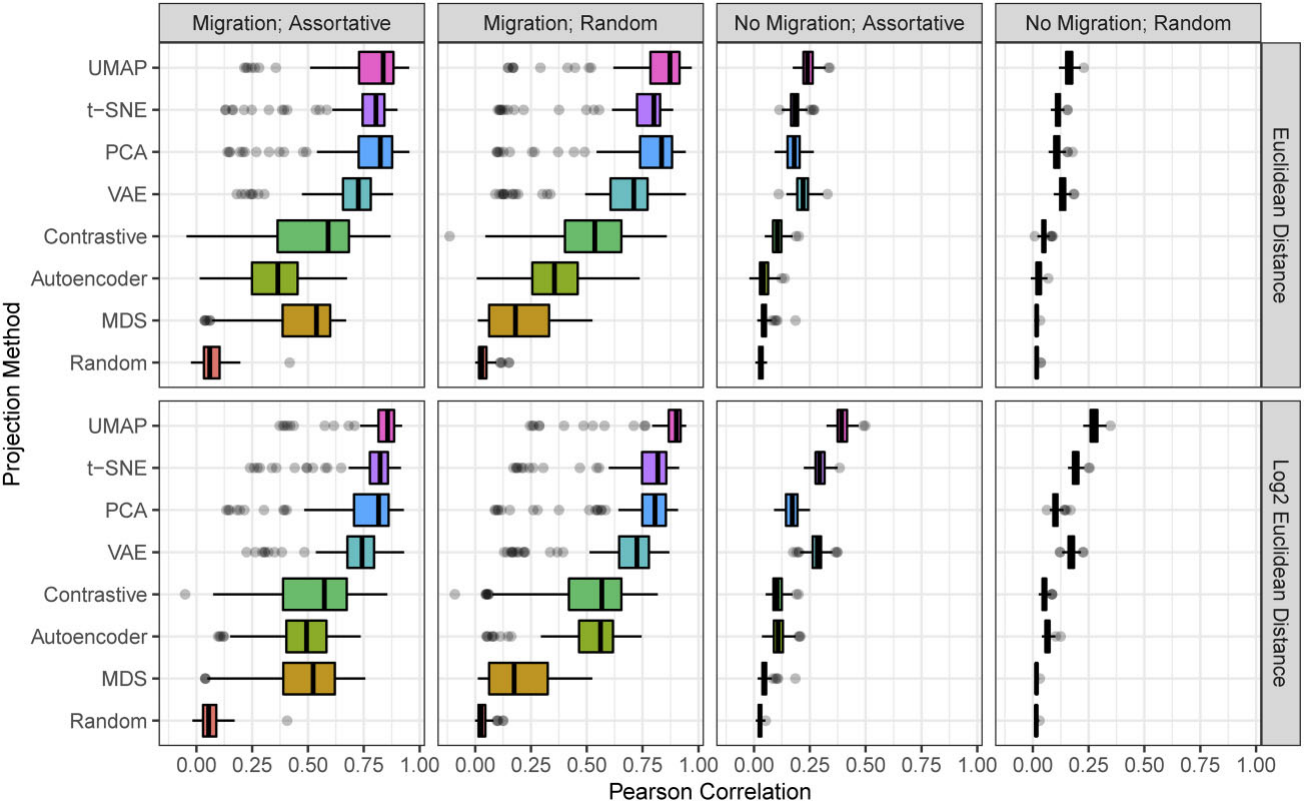
Pearson correlation (*r*) between the Euclidean distance (*D*; top row) and ${\;\text{log}\;}_{2}$ of the Euclidean distance (${\;\text{log}\;}_{2}(D)$; bottom row) in the visualization and the known pedigree distance in simulations with 8 projection methods. Each simulation is repeated 100 times.

For the simulations, the superscript letters are the compact letter display for the Tukey least significant difference test (shown in Fig. 3). The standard deviations from the simulated samples are shown in parentheses following the correlation coefficient. Highest correlation values for each dataset are bolded for readability.

**Fig. 4.**
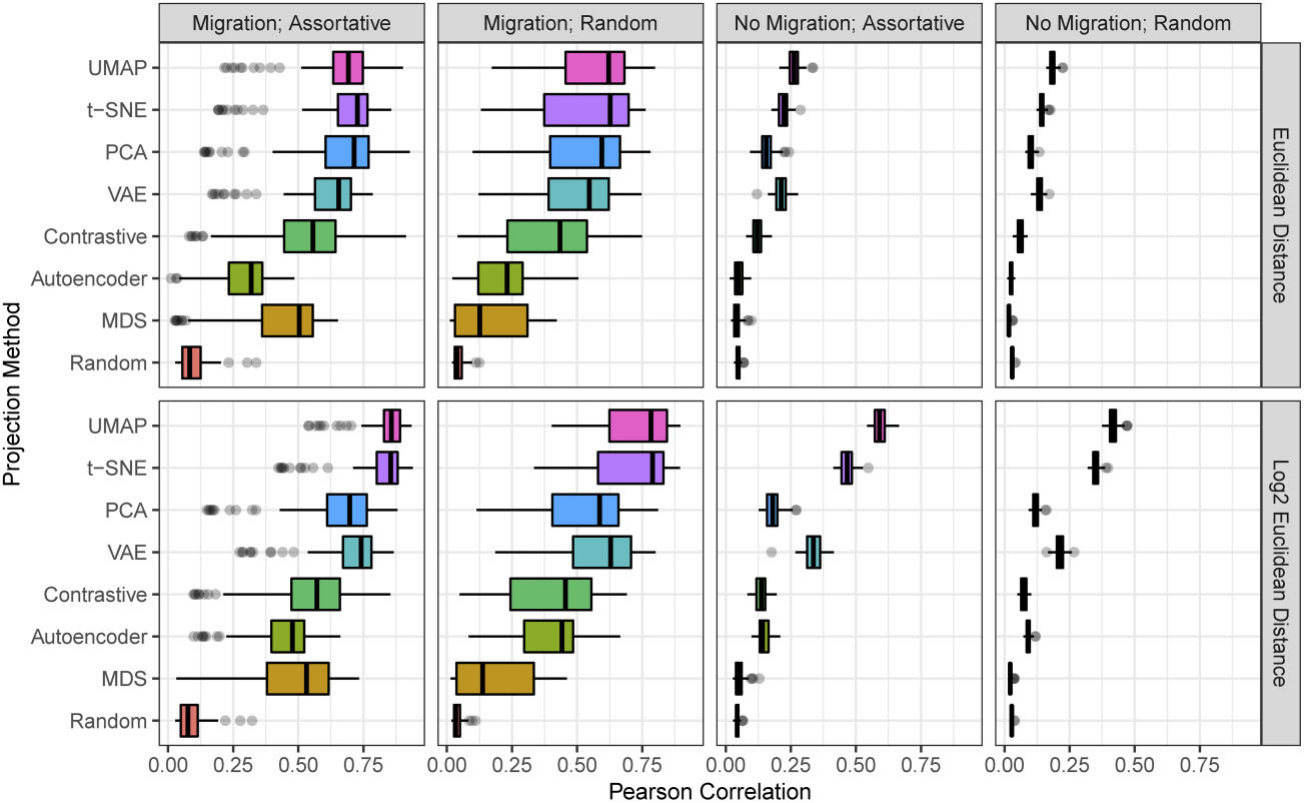
Pearson correlation (*r*) between the Euclidean distance (*D*; top row) and ${\;\text{log}\;}_{2}$ of the Euclidean distance (${\;\text{log}\;}_{2}(D)$; bottom row) in the visualization and the known IBD distance in simulations with 8 projection methods. Each simulation is repeated 100 times.

On the basis that the pedigree distance ground truth could be affected by populations containing inbreeding, we repeat all of the analyses using the IBD matrices as the ground truth. We report Pearson’s correlation between the Euclidean distance (or ${\;\text{log}\;}_{2}$ of this distance) in the visualization and the inverse of the IBD similarity. These results are shown in Table 2. The correlation between the ground-truth distances and the Euclidean distance in the ambient marker space is shown in Table 3 for reference. Detailed results for the simulation experiments are shown in Figure 3 and Figure 4.

**Table 2.** Pearson correlation (*r*) between the Euclidean distance, *D*, and ${\;\text{log}\;}_{2}$ of the Euclidean distance, ${\;\text{log}\;}_{2}(D)$, in the visualization and the IBD distance.

Distance metric	Model	Strawberry	Pig	Soay sheep	Florida Scrub-Jay	Migration		No migration	
						Random	Assortative	Random	Assortative
*D*	UMAP	0.51	0.37	0.18	0.13	**0.56** (± 0.18)^*d*^	0.67 (± 0.14)^*e*^	**0.18** (± 0.01)^*g*^	**0.26** (± 0.02)^*e*^
	t-SNE	0.42	**0.42**	**0.21**	**0.21**	0.54 (± 0.20)^*d*^	**0.70** (± 0.16)^*e*^	0.14 (± 0.01)^*f*^	0.22 (± 0.02)^*d*^
	PCA	**0.57**	0.29	0.03	0.08	0.52 (± 0.20)^*d*^	0.65 (± 0.18)^*e*^	0.10 (± 0.01)^*d*^	0.16 (± 0.02)^*c*^
	VAE	0.41	0.34	0.12	0.08	0.49 (± 0.18)^*d*^	0.61 (± 0.15)^*e*^	0.13 (± 0.01)^*e*^	0.21 (± 0.03)^*d*^
	Contrastive	0.47	0.29	0.03	0.10	0.38 (± 0.19)^*c*^	0.52 (± 0.18)^*d*^	0.06 (± 0.01)^*c*^	0.12 (± 0.02)^*b*^
	AE	0.31	0.13	0.00	0.10	0.22 (± 0.12)^*b*^	0.29 (± 0.11)^*b*^	0.03 (± 0.01)^*b*^	0.05 (± 0.02)^*a*^
	MDS	0.32	0.16	0.04	0.10	0.17 (± 0.13)^*b*^	0.44 (± 0.17)^*c*^	0.02 (± 0.00)^*a*^	0.04 (± 0.02)^*a*^
	Random	0.10	0.05	0.03	0.10	0.05 (± 0.02)^*a*^	0.10 (± 0.06)^*a*^	0.03 (± 0.00)^*b*^	0.05 (± 0.01)^*a*^
${\;\text{log}\;}_{2}(D)$	UMAP	0.48	0.51	0.25	0.31	**0.73** (± 0.15)^*e*^	**0.84** (± 0.09)^*e*^	**0.42** (± 0.02)^*g*^	**0.59** (± 0.03)^*f*^
	t-SNE	0.42	**0.56**	**0.32**	**0.43**	0.70 (± 0.17)^*e*^	0.81 (± 0.12)^*e*^	0.35 (± 0.01)^*f*^	0.47 (± 0.03)^*e*^
	PCA	**0.52**	0.28	0.04	0.14	0.52 (± 0.19)^*d*^	0.65 (± 0.17)^*d*^	0.12 (± 0.01)^*d*^	0.18 (± 0.03)^*c*^
	VAE	0.37	0.38	0.17	0.04	0.57 (± 0.18)^*d*^	0.69 (± 0.14)^*d*^	0.21 (± 0.02)^*e*^	0.33 (± 0.04)^*d*^
	Contrastive	0.37	0.27	0.04	0.16	0.41 (± 0.19)^*c*^	0.54 (± 0.18)^*c*^	0.07 (± 0.01)^*c*^	0.14 (± 0.02)^*b*^
	AE	0.35	0.20	0.06	0.20	0.39 (± 0.15)^*c*^	0.44 (± 0.12)^*b*^	0.09 (± 0.01)^*b*^	0.15 (± 0.02)^*b*^
	MDS	0.31	0.20	0.06	0.07	0.18 (± 0.14)^*b*^	0.47 (± 0.19)^*b*^	0.02 (± 0.00)^*a*^	0.05 (± 0.02)^*a*^
	Random	0.05	0.01	0.01	−0.02	0.04 (± 0.02)^*a*^	0.09 (± 0.05)^*a*^	0.03 (± 0.00)^*a*^	0.04 (± 0.01)^*a*^

For the simulations, the superscript letters are the compact letter display for the Tukey least significant difference test (shown in Fig. 4). The standard deviations from the simulated samples are shown in parentheses following the correlation coefficient. Highest correlation values for each dataset are bolded for readability.

**Table 3.** Correlation between the Euclidean distance (*D*) and ${\;\text{log}\;}_{2}$ of the Euclidean distance (${\;\text{log}\;}_{2}(D)$) in the marker space and the ground-truth ancestral distance for the natural datasets.

Dataset	Pedigree distance		IBD distance	
	*D*	log_2_*D*	*D*	log_2_*D*
Strawberry	0.79	0.78	0.62	0.51
Pig	0.70	0.69	0.66	0.60
Soay sheep	0.76	0.74	0.74	0.58
Florida Scrub-Jay	0.15	0.08	0.12	0.16

### Comparison of linear and nonlinear methods

Our evaluations show that nonlinear methods are often superior alternatives to PCA for visualizing population structure. t-SNE or UMAP outperformed PCA in every dataset, with the exception of strawberry. The strength of PCA in the strawberry example may be due to ascertainment bias in genotyping arrays, depth and complexity of the pedigree records, strength of selection between different subgroups, or the overrepresentation of modern *Fragaria × ananassa* in the database. PCA was outperformed by 1 or more of the neural network methods (AE, VAE, and contrastive) in 5 of the 8 datasets when linear distance was assumed and 4 of the 8 when exponential distance was assumed, demonstrating that there is little incentive to use these techniques over PCA as a standard choice. Of the neural network-based methods, VAE and contrastive learning alternated as the best performing in the real datasets, although the VAE was superior in the experiments with simulated data. MDS was the worst-performing method in our tests, coming in last in almost all of the experiments. All of the methods did consistently perform better than the random projection, indicating that they do capture significant information about ancestry.

The experiments using the pedigree path distance (Table 1) and the IBD similarity (Table 2) as ground truth are similar in the trend of their results. The pedigree distance resulted in generally higher correlation values on average. Whether using pedigree distance or IBD similarity, some datasets provided generally low correlation values. These are the Florida Scrub-Jay population, and the simulations without migration. These universally low correlation values could be attributed to true or artificial uniformity in the pedigree distances.

The empirical datasets strongly differ in their median depth (distance)—the median depth in the Scrub-Jay dataset is 2 generations, while the Soay sheep, pig, and strawberry have median depths of 5, 7, and 7, respectively (Tables 1 and 2). When the majority of individuals included in a pedigree are assumed to be unrelated (no connectivity), the distance between individuals in inestimable. A moderate level of depth, e.g. median depth ≥5, is likely a necessity to eliminate the false uniformity of relationships when nearly all individuals are assumed to be unrelated. In the ideal scenario for our analyses, all pedigree connections would be known and all individuals are a known distance from every other individual.

Similarly, the “no migration” simulations yielded small correlation coefficients between the projection distances and the pedigree distances (Tables 1 and 2). This is likely because the pedigree distances are uniform and the projections are likely sensitive to initial conditions and may also prioritize global over local structure. When there are only local structure data, the projections are simply prone to weaker performance compared to when there is discernible global structure. Even when there is no global structure, there are significant differences in the performance of the different metrics with t-SNE and UMAP systematically outperforming other included methods.

In addition to the quantitative results, the visualizations in Fig. 1c of the simulated population in Fig. 1b illustrate some key differences between the methods. The ground-truth plot shows that there are 7 major clusters of subpopulations, with some broken down further into multiple smaller subpopulations. Only some of these clusters are captured with PCA, as the first 2 principal components are insufficient to differentiate between some of the more closely related subpopulations. The AE shows its characteristic “smearing” of points in the space, while clusters are vaguely visible. It is apparent that the AE does not consider pairwise Euclidean distance to be important—the heads of each cluster are closer to each other than to the points in their tails. The VAE compacts clusters better than the AE but still tends to pull points toward the mode of the prior distribution at the origin.

Meanwhile, the use of cosine distance in the contrastive method creates a ring-like structure, as the positions of the points are based on the angle they make with the origin. t-SNE and UMAP show the major clusters clearly, although UMAP clusters individuals more tightly within each group, while t-SNE prefers to spread each cluster so individual points can be seen more clearly. This difference may account for the advantage UMAP holds over t-SNE in the quantitative results. Hyperparameters could also be tuned to decrease the tightness of the clusters in UMAP, if larger clusters are preferable for visualization reasons. Compared to the other techniques, the advantages of t-SNE and UMAP are clearly demonstrated here. These methods do not lose important information like PCA, which collapses several of the subpopulations together, and they respect pairwise Euclidean distances between individuals better than the neural network-based methods.

### On the success or failure of methods

It is important to note that the pedigree distance metric we develop here only measures how closely a visualization matches the viewer’s expectations of what it shows—that is to what degree distances between points are indicative of differences in ancestry. The individual methods succeed or fail on this metric by incidence, not by design. For example, a method such as UMAP does not guarantee to preserve pairwise Euclidean distances between every pair of points, but rather, it seems to portray this incidentally in our experiments. Conversely, a method such as contrastive embedding learning is not a generally weak method for learning good representations of data—it only fails to portray what we expect an embedding method used in this domain to show.

### Limitations

Although we have attempted to quantify the “accuracy” of different visualizations with as much objectivity as possible, it remains true that visualization is inherently subjective. Even given these evaluations, practitioners may still prefer one visualization over another based on how it portrays certain known characteristics of the population. Therefore, our results only provide additional information for decision-making based on a particular set of goals, not a blanket directive for which methods should be used.

All of the quantitative results reported here are associated with a particular set of assumptions. We find these assumptions to be reasonable—that distance between individuals should correlate with distance in ancestry—but not necessarily universal. For example, our evaluation metric assumes that this correlation should be linear (or linear following a log transform). Some researchers may prefer a method that exaggerates some types of distances, while minimizing others. This is not a problem, so long as the audience is aware of how the visualization should be interpreted. Other researchers may be either more or less tolerant to outliers than is reflected by our choice of Pearson’s *r* as the evaluation metric.

One weakness of the pedigree distance metric in natural datasets is that it does not measure the accuracy of distances between completely unrelated subpopulations, as there is no known pedigree relationship between these individuals. For example, a dataset containing 2 such subpopulations will attain the same performance on the pedigree distance metric whether the subpopulations are shown as completely separate or overlapping. This creates a possible bias in evaluation, as pedigree relationships are more likely to exist for closely related individuals—that is the ground truth distances are more likely to exist between samples that reside in the same local neighborhood. We have addressed this by using simulations, where ancestral relationships between every pair of individuals are known. When taking these distant relationships into account using the simulated data, we see that the same trends in performance hold for all of the methods, suggesting that this effect is likely not a significant drawback for our evaluations on the natural datasets.

We believe that our simulations (while not representative of every scenario) do capture general patterns of what we would expect in situations with selection and/or migration, as we show that the allele frequency spectrum shifts much more dramatically in the selected populations than in the randomly mated populations, regardless of migration (Figure 2). However, our simulations start out with uniform allele frequencies across sites. This may be simplistic, but we feel it captures the general trend of segregating sites in populations undergoing random and assortative mating.

## Conclusions

To evaluate which embedding methods most accurately portray differences in ancestry, we have performed quantitative and qualitative analyses. Our analyses suggest that graph-based algorithms such as t-SNE and UMAP may provide visualizations of population structure where differences in ancestry are portrayed more accurately than with other dimensionality reduction methods, such as PCA or neural network-based methods. The results are consistent across real-world datasets as well as simulations, and for both random mating and assortative mating (breeding) populations with and without subpopulation structure. The results are also consistent whether evaluated on the basis of either linear or exponential distance with depth of ancestry, and using either the pedigree distance or the IBD matrix as the ground truth. Our visualizations illustrate where nonlinear methods can improve on the embeddings provided by PCA, as well as how neural network-based methods such as AEs and VAEs fail in ways, which are anticipated by theory.

## Data availability

Data are publicly available from the sources referenced by their authors. Code for reproducing the experiments is available at https://github.com/jubbens/popmodels.
